# Comparison of safety of lecanemab and donanemab: a real-world disproportionality analysis using the FDA adverse event reporting system

**DOI:** 10.3389/fphar.2026.1868789

**Published:** 2026-06-22

**Authors:** Xiuping Feng, Shangqing Bi, Chaoqiong Shi, Qingqing Chang, Jiaming Zhang, Yiyang Zhuang

**Affiliations:** 1 The Seventh Clinical College of Guangzhou University of Chinese Medicine, Shenzhen, Guangdong, China; 2 Shenzhen Bao’an Chinese Medicine Hospital, Shenzhen, Guangdong, China

**Keywords:** adverse events, Alzheimer’s disease, disproportionality analysis, donanemab, FAERS, lecanemab, pharmacovigilance

## Abstract

**Background:**

Lecanemab and donanemab are anti-amyloid-β (Aβ) monoclonal antibodies recently approved for the treatment of Alzheimer’s disease (AD). Although both agents have demonstrated therapeutic potential, their post-marketing adverse event reporting profiles remain insufficiently characterized and compared in spontaneous reporting systems. This study aimed to systematically compare adverse event signals associated with these two drugs using the FDA Adverse Event Reporting System database, with sex-stratified and sensitivity analyses performed to support the main findings.

**Methods:**

FAERS reports up to the fourth quarter of 2025 were retrospectively analyzed. Reports in which lecanemab or donanemab was recorded as the primary suspect drug were included. AEs were classified by system organ classes (SOCs) and preferred terms (PTs) according to the Medical Dictionary for Regulatory Activities (MedDRA). Signal detection was performed using four algorithms: reporting odds ratio (ROR), proportional reporting ratio (PRR), Bayesian confidence propagation neural network (BCPNN), and multi-item gamma Poisson shrinker (MGPS). Sex-stratified analysis, sensitivity analysis after excluding reports involving concomitant medications, and time-to-onset (TTO) analysis based on the Weibull shape parameter model were also conducted. False discovery rate (FDR) correction was applied to analyses involving multiple P values.

**Results:**

A total of 3,640 AE reports were identified, including 2,602 for lecanemab and 1,038 for donanemab. Nervous system disorders was the most frequently reported SOC and the only SOC meeting the positivity criteria across all four algorithms for both drugs. At the PT level, the frequently reported events for both drugs were mainly concentrated in amyloid-related imaging abnormality (ARIA)-related events, including ARIA with oedema/effusion (ARIA-E) and ARIA with microhaemorrhages/haemosiderin deposition (ARIA-H), headache, and infusion-related reactions. The strongest disproportionality reporting signals were mainly observed for ARIA-related preferred terms and cerebral microhaemorrhage. In separate FAERS-based disproportionality analyses, lecanemab showed stronger disproportionality reporting signals for ARIA-H, whereas donanemab showed stronger disproportionality reporting signals for ARIA-E. In addition, drug-specific PT signal distributions differed between the two agents. Several potential novel PT signals were also identified. Sex-stratified analysis suggested differences in the distribution of certain PT signals between female and male reports. Sensitivity analysis supported the stability of most core signals. The median TTO was 46 days for lecanemab and 31 days for donanemab, and Weibull analysis suggested different temporal patterns of AE occurrence.

**Conclusion:**

Using four signal detection algorithms, this study compared real-world adverse event reporting profiles associated with lecanemab and donanemab. The two drugs showed both shared and distinct adverse event reporting profiles, particularly in ARIA subtype-related disproportionality signals, drug-specific PT signals, potential novel reporting signals, sex-stratified reporting patterns, and TTO characteristics. These findings provide pharmacovigilance evidence for targeted safety monitoring and hypothesis generation, but should not be interpreted as causal associations or true incidence estimates.

## 1 Introduction

Alzheimer’s disease (AD) is the most prevalent neurodegenerative disorder and is characterized by progressive cognitive decline (Ballard et al., 2011; Ryan et al., 2019). According to the World Health Organization, more than 55 million people worldwide are currently living with dementia, and this number is projected to reach 139 million by 2050 (Long et al., 2023). Current therapeutic strategies are limited to symptomatic relief and do not alter the underlying disease progression (Passeri et al., 2022). With the growing clinical demand, disease-modifying therapies targeting key pathological mechanisms have emerged as a major focus in AD research.

The deposition of amyloid-β (Aβ) is a central hypothesis in the pathogenesis of AD (Ciuperca et al., 2024). Based on this hypothesis, anti-Aβ monoclonal antibodies have become a major focus of recent therapeutic development. Lecanemab and donanemab, as representative agents in this class, were approved by the United States Food and Drug Administration (FDA) in July 2023 and July 2024, respectively, for the treatment of early AD (FDA, 2023; FDA, 2024). Lecanemab is a humanized immunoglobulin G1 (IgG1) monoclonal antibody targeting soluble Aβ protofibrils (Alkhalifa et al., 2025), whereas donanemab is a humanized IgG1 monoclonal antibody targeting deposited, modified Aβ plaques (Kovacik et al., 2025). Both agents promote cerebral Aβ clearance, exert disease-modifying effects, and have demonstrated efficacy in slowing cognitive decline in pivotal clinical trials (Villain et al., 2025; Kang, 2024).

However, these therapeutic benefits are accompanied by non-negligible safety concerns. Although both agents belong to the class of anti-Aβ monoclonal antibodies, they differ in target epitopes, plaque-clearing patterns, and dosing strategies. Accordingly, the associated adverse drug events (ADEs) may show both shared and drug-specific features. Pivotal clinical trials have shown that both drugs are commonly associated with amyloid-related imaging abnormalities (ARIA) and infusion-related reactions. ARIA is an umbrella term for magnetic resonance imaging (MRI)-detected abnormalities associated with anti-amyloid-β monoclonal antibody therapy. It mainly includes two subtypes: ARIA with oedema/effusion (ARIA-E), characterized by vasogenic oedema or sulcal effusion, and ARIA with haemorrhage/haemosiderin deposition (ARIA-H), characterized by cerebral microhaemorrhage, macrohaemorrhage, or superficial siderosis. Given that ARIA represents one of the most clinically important safety concerns during anti-Aβ monoclonal antibody therapy, clarifying its subtype-specific reporting patterns may help inform post-marketing pharmacovigilance monitoring. At the same time, differences in adverse events (AEs) between the two drugs have also attracted increasing attention (Cho et al., 2023; Sims et al., 2023). Given the relatively recent approval of both agents, current understanding of their associated adverse events remains at an early stage (Kaur et al., 2024; Høilund-Carlsen et al., 2024), and systematic comparative evidence based on real-world data is still limited. Therefore, characterizing and comparing the post-marketing adverse event reporting patterns of lecanemab and donanemab may provide useful pharmacovigilance evidence for optimizing clinical monitoring.

Clinical trials are constrained by strict inclusion and exclusion criteria, limited sample sizes, and relatively short follow-up durations, and thus may not fully capture the complete safety profile in real-world populations, particularly in patients receiving concomitant medications. The Food and Drug Administration’s Adverse Event Reporting System (FAERS) is one of the most widely used spontaneous reporting databases in pharmacovigilance and provides important data for post-marketing signal detection (Rong et al., 2024; Jiang et al., 2024). Although previous pharmacovigilance studies have separately investigated adverse events associated with lecanemab or donanemab (Li et al., 2025; Li, 2026), evidence describing the similarities and differences in their post-marketing safety profiles remains limited. Therefore, further characterization of AE reporting patterns and disproportionality signals for these two anti-amyloid monoclonal antibodies is of practical importance for pharmacovigilance monitoring. To address this gap, the present study used the FAERS database to systematically evaluate post-marketing adverse events associated with lecanemab and donanemab. Sex-stratified subgroup analyses and sensitivity analyses excluding major concomitant medications were further performed to complement and support the main findings. This study may provide real-world evidence to support clinical risk monitoring and safety evaluation of these two drugs.

## 2 Materials and methods

### 2.1 Data source

**FIGURE 1:**
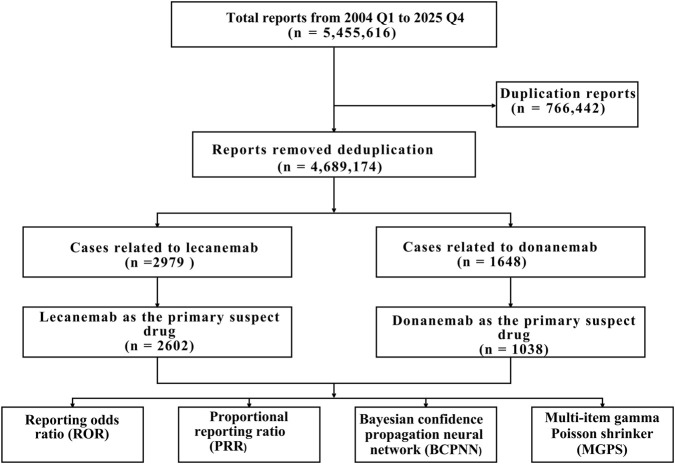
Data filtering flowchart.

This study was based on publicly available quarterly FAERS data and included all available reports up to the fourth quarter of 2025. The FAERS database mainly comprises core data files containing patient demographic information (DEMO), drug information (DRUG), adverse event information (REAC), patient outcome information (OUTC), reporter source information (RPSR), therapy date information (THER), and drug indication information (INDI). The structure and content of these tables follow the International Council for Harmonisation (ICH) safety reporting guidelines, and AEs are coded using the Medical Dictionary for Regulatory Activities (MedDRA). Duplicate reports were removed on the basis of case ID and primary ID, and all analyses were conducted using deduplicated reports. Because this study was based exclusively on publicly available, de-identified FAERS data, it did not involve personal privacy information, and ethical approval and informed consent were not required. The data processing workflow is shown in Figure 1.

### 2.2 Data extraction and processing

First, quarterly data files were vertically merged and linked across different tables using primaryid and caseid to integrate complete information for each case. In the DRUG file, both generic names (lecanemab and donanemab) and brand names (Leqembi and Kisunla) were used as search terms, and only records with the drug role coded as “PS” (primary suspect) were retained to focus as much as possible on AEs strongly associated with the target drugs. Deduplication followed the FDA-recommended procedure: when multiple reports shared the same caseid, the record with the most recent FDA_DT (FDA receipt date) was retained; if both caseid and FDA_DT were identical, the record with the largest primaryid value was retained as the unique report for final analysis. All extracted AEs were standardized using MedDRA version 26.0. In the analysis, AEs were described at the preferred term (PT) level and were further aggregated into the system organ class (SOC) level to evaluate organ system-level distributions (Brown, , 2004; Tieu and Breder, 2018).

Clinical characteristics, including sex, reporting country, reporter occupation, and reporting year, were described for all included reports. In addition, this study analyzed the time-to-onset (TTO) of lecanemab and donanemab-associated events. TTO was defined as the interval in days between the event date (EVENT_DT) and the treatment start date (START_DT). Reports with missing dates, non-calculable intervals, or obvious logical errors were excluded. Because a single case may appear repeatedly due to multiple PTs, deduplication by primaryid was performed for the TTO analysis to avoid repeated counting of the same case, and only one TTO value was retained per case for subsequent statistical analysis. If multiple calculable EVENT_DT values were present under the same primaryid, the TTO corresponding to the earliest event date was retained. Based on the descriptive TTO analysis, the Weibull shape parameter (WSP) model was further used to evaluate temporal reporting patterns of AEs associated with the two drugs. The model includes a scale parameter (α) and a shape parameter (β). When β < 1 and the upper limit of its 95% confidence interval (CI) is < 1, the pattern indicates early failure, suggesting that the event risk gradually decreases over time. When the 95% CI of β includes 1, the pattern indicates random failure. When β > 1 and the lower limit of its 95% CI is > 1, the pattern indicates wear-out failure, suggesting that the event risk gradually increases over time. Because FAERS is a spontaneous reporting database rather than a longitudinal follow-up cohort, information on censoring due to death, treatment discontinuation, loss to follow-up, or continued event-free exposure is not systematically recorded. Therefore, censored observations could not be identified or incorporated into the TTO analysis. The TTO analysis was restricted to reports with valid treatment start dates and event dates, and reports with missing, invalid, or non-calculable TTO values were excluded. Accordingly, no censoring-based survival analysis was performed.

### 2.3 Signal detection

Disproportionality analysis (DPA) is a commonly used approach in spontaneous reporting databases to identify disproportionate reporting signals between drugs and AEs (Caster et al., 2020). In this study, lecanemab and donanemab were treated as the target drugs, and all reports involving other drugs in the FAERS database during the same study period were used as the reference group. Accordingly, 2 × 2 contingency tables were constructed (Table 1) to calculate four signal indicators, namely, the reporting odds ratio (ROR), proportional reporting ratio (PRR), Bayesian confidence propagation neural network (BCPNN; expressed as IC025), and multi-item gamma Poisson shrinker (MGPS; expressed as EBGM05). ROR and PRR are frequentist methods that are easy to calculate and highly sensitive (Rothman et al., 2004; Evans et al., 2001), but they may yield false-positive findings when event counts are limited (Wu et al., 2023). BCPNN and MGPS are Bayesian methods that are generally more stable for low-frequency events (Bate et al., 1998; Kubota et al., 2004). To improve the robustness of signal detection and reduce potential false positives arising from a single algorithm, only events meeting the positive thresholds of all four algorithms simultaneously were considered positive signals (Kong et al., 2025). The formulas and positivity criteria for the four indicators are shown in Table 2.

**TABLE 1:** Four-grid table for disproportionality analysis.

AE category	Target drug	Other drugs	Total
AE of interest	a	b	a+b
Other AEs	c	d	c + d
Total	a+c	b + d	a + b + c + d

The variables are defined as follows: a, the number of reports for the target drug and the adverse event of interest; b, the number of reports for other drugs and the adverse event of interest; c, the number of reports for the target drug and all other adverse events; d, the number of reports for all other drugs and all other adverse events.

**TABLE 2:** Summary of the main algorithms used for signal detection.

Method	Formula	Threshold
ROR	$\text{ROR}=\frac{\left(a/c\right)}{\left(b/d\right)}=\frac{\text{ad}}{\text{bc}}$ $\text{SE}\left(\text{lnROR}\right)=\sqrt{\left(\frac{1}{a}+\frac{1}{b}+\frac{1}{c}+\frac{1}{d}\right)}$	a≥3 and 95% CI (lower limit) > 1
PRR	$\text{PRR}=\frac{a/\left(a+b\right)}{c/\left(c+d\right)}$ $\text{SE}\left(\text{lnPRR}\right)=\sqrt{\frac{1}{a}‐\frac{1}{a+b}+\frac{1}{c}‐\frac{1}{c+d}}$ $95\%\text{ CI}=e^{\ln\left(\text{PRR}\right)\pm 1.96\sqrt{\frac{1}{a}‐\frac{1}{a+b}+\frac{1}{c}‐\frac{1}{c+d}}}$	a≥3 and PRR ≥2, χ^2^ ≥ 4
BCPNN	$\text{IC}=\log_{2}\frac{p\left(x,y\right)}{p\left(x\right)p\left(y\right)}=\log_{2}\frac{a\left(a+b+c+d\right)}{\left(a+b\right)\left(a+c\right)}E\left(\text{IC}\right)=\log_{2}\frac{\left(a+\gamma 11\right)\left(a+b+c+d+\alpha \right)\left(a+b+c+d+\beta \right)}{\left(a+b+c+d+\gamma \right)\left(a+b+\alpha 1\right)\left(a+c+\beta 1\right)}$ $V\left(\text{IC}\right)=\frac{1}{{\left(\ln2\right)}^{2}}\left\{[\frac{\left(a+b+c+d\right)‐a+\gamma ‐\gamma 11}{\left(a+\gamma 11\right)\left(1+a+b+c+d+\gamma \right)}\right]+\left[\frac{\left(a+b+c+d\right)‐\left(a+b\right)+\alpha ‐\alpha 1}{\left(a+b+\alpha 1\right)\left(1+a+b+c+d+\alpha \right)}\right]\left.+\left[\frac{\left(a+b+c+d\right)‐\left(a+c\right)+\beta ‐\beta 1}{\left(a+c+\beta 1\right)\left(1+a+b+c+d+\beta \right)}\right]\right\}$ $\gamma =\gamma 11\frac{\left(a+b+c+d+\alpha \right)\left(a+b+c+d+\beta \right)}{\left(a+b+\alpha 1\right)\left(a+c+\beta 1\right)}$ $\text{IC}‐2\text{SD}=E\left(\text{IC}\right)‐2\sqrt{V\left(\text{IC}\right)}$	IC025 > 0
EBGM	$\text{EBGM}=\frac{a\left(a+b+c+d\right)}{\left(a+c\right)\left(a+b\right)}$ $95\%\text{ CI}=e^{\ln\left(\text{EBGM}\right)\pm 1.96\sqrt{\frac{1}{a}+\frac{1}{b}+\frac{1}{c}+\frac{1}{d}}}$	EBGM05 > 2

Abbreviations: ROR, reporting odds ratio; PRR, proportional reporting ratio; BCPNN, bayesian confidence propagation neural network; MGPS, multi-item gamma Poisson shrinker; IC, information component; EBGM, empirical Bayes geometric mean; CI, confidence interval; χ2, chi-squared; IC025, lower limit of 95% two-sided CI, of the IC; EBGM05, lower limit of 95% one-sided CI, of EBGM.

To further evaluate the level of prior evidence supporting positive PTs, this study performed evidence stratification for PT signals that met the positivity criteria of all four algorithms by integrating FDA drug labels, publicly available safety results from pivotal clinical trials, and previous post-marketing literature. FDA labels were retrieved from Drugs@FDA, and pivotal trial and post-marketing evidence was searched in PubMed through 31 December 2025. According to the level of prior evidence support, positive PTs were classified into three categories: (1) PTs listed in the FDA label, marked with #; (2) PTs reported in pivotal clinical trials or previous literature but not explicitly listed in the label, marked with %; (3) PTs not clearly reported in the label, pivotal clinical trials, or previous literature, defined as “potential novel signals” and marked with *. The stratification results were independently reviewed by two researchers, and disagreements were resolved through discussion with a third reviewer. The stratification results were used only to describe and discuss signal novelty in the present study and were not intended for causal inference.

### 2.4 Subgroup and sensitivity analyses

To explore potential sex-related differences and assess the robustness of the main findings, two additional analyses were conducted: sex-stratified analysis and sensitivity analysis based on concomitant medications. In the sex-stratified analysis, PT-level signal detection was repeated separately in male and female reports, and the direction and strength of RORs were compared between the two subgroups. In the sensitivity analysis, reports involving the top 10 most frequently reported concomitant medications were excluded, and signal detection was repeated to evaluate whether the main reporting signals remained detectable after reducing the influence of common co-medications. The top 10 concomitant medications were selected because they represented the most frequently reported co-medications and were therefore considered most likely to influence the overall reporting signal distribution.

### 2.5 Multiple-comparison adjustment

Given the exploratory nature of this pharmacovigilance study, multiple comparisons were performed across SOC-level, PT-level, subgroup, and sensitivity analyses. To reduce the probability of chance findings caused by multiple testing, the Benjamini–Hochberg false discovery rate (FDR) procedure was applied to analyses in which multiple P values were generated. FDR-adjusted P values <0.05 were considered statistically significant. The FDR correction was used only as a supplementary statistical adjustment and did not replace the predefined disproportionality criteria for signal detection.

## 3 Results

### 3.1 Descriptive characteristics

**FIGURE 2:**
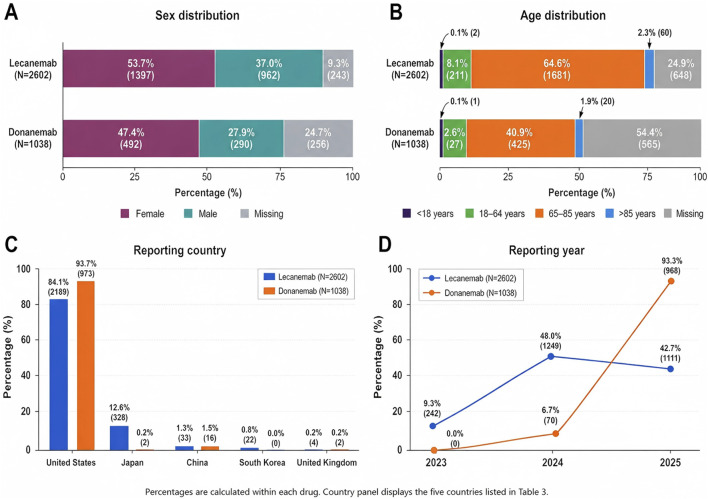
Distribution of reporting characteristics for lecanemab and donanemab in the FAERS database. **(A)** Distribution by sex; **(B)** Distribution by age; **(C)** Distribution by reporting country; **(D)** Distribution by reporting year.

As shown in Table 3, a total of 3,640 AE reports were identified, including 2,602 reports associated with lecanemab (71.5%) and 1,038 reports associated with donanemab (28.5%). The numbers of reports from female and male patients were 1,889 (51.9%) and 1,252 (34.4%), respectively. Consumers accounted for the largest proportion of reporters (1,626 reports, 44.7%), followed by physicians (1,065 reports, 29.3%). Most reports originated from the United States (3,162 reports, 86.9%). Lecanemab reports were mainly concentrated in 2024 and 2025, whereas donanemab reports were highly concentrated in 2025. The detailed distributions of sex, age, reporting country, and reporting year for the two drugs are shown in Figure 2.

**TABLE 3:** Descriptive characteristics of FAERS reports for lecanemab and donanemab.

Description	Lecanemab	Donanemab	Overall
Number of reports	(N = 2,602)	(N = 1,038)	(N = 3,640)
Sex			
Female	1,397 (53.7%)	492 (47.4%)	1889 (51.9%)
Male	962 (37.0%)	290 (27.9%)	1,252 (34.4%)
Missing	243 (9.3%)	256 (24.7%)	499 (13.7%)
Age (years)			
<18	2 (0.1%)	1 (0.1%)	3 (0.1%)
18–64	211 (8.1%)	27 (2.6%)	238 (6.5%)
65–85	1,681 (64.6%)	425 (40.9%)	2,106 (57.9%)
>85	60 (2.3%)	20 (1.9%)	80 (2.2%)
Missing	648 (24.9%)	565 (54.4%)	1,213 (33.3%)
Report types			
Consumer	683 (26.2%)	943 (90.8%)	1,626 (44.7%)
Health professional	371 (14.3%)	52 (5.0%)	423 (11.6%)
Pharmacist	49 (1.9%)	7 (0.7%)	56 (1.5%)
Physician	1,049 (40.3%)	16 (1.5%)	1,065 (29.3%)
Missing	450 (17.3%)	20 (1.9%)	470 (12.9%)
Report countries			
United States	2,189 (84.1%)	973 (93.7%)	3,162 (86.9%)
Japan	328 (12.6%)	2 (0.2%)	330 (9.1%)
China	33 (1.3%)	16 (1.5%)	49 (1.3%)
South Korea	22 (0.8%)	0 (0.0%)	22 (0.6%)
United Kingdom	4 (0.2%)	2 (0.2%)	6 (0.2%)
Report years			
2023	242 (9.3%)	0 (0.0%)	242 (6.6%)
2024	1,249 (48.0%)	70 (6.7%)	1,319 (36.2%)
2025	1,111 (42.7%)	968 (93.3%)	2079 (57.1%)

### 3.2 SOCs involved in positive signals

Tables 4, 5 list the top 10 SOCs ranked by report frequency and their corresponding signal strengths for lecanemab and donanemab in the FAERS database. For both drugs, nervous system disorders was the most frequently reported SOC and the only SOC that met the positivity criteria across all four algorithms. In addition, general disorders and administration site conditions, injury, poisoning and procedural complications, and psychiatric disorders were frequently reported for both drugs, although they did not meet the positivity criteria across all four algorithms simultaneously. After FDR correction, the SOC-level results for both lecanemab and donanemab remained generally unchanged, and no SOC with nominal statistical significance lost significance after correction.

**TABLE 4:** Top 10 SOCs for lecanemab by report frequency in FAERS.

SOC	Number of reports	ROR (95% CI)	PRR (χ^2^)	EBGM (EBGM05)	IC (IC025)
Nervous system disorders	1867	8.3 (7.83–8.79)	5.45 (7,292.46)	5.44 (5.13)	2.44 (2.36)
General disorders and administration site conditions	902	1.09 (1.01–1.17)	1.07 (5.01)	1.07 (1.00)	0.1 (−0.01)
Injury, poisoning and procedural complications	337	0.48 (0.43–0.53)	0.51 (179.92)	0.51 (0.46)	−0.96 (−1.12)
Psychiatric disorders	318	1.46 (1.30–1.63)	1.43 (42.50)	1.43 (1.27)	0.51 (0.34)
Gastrointestinal disorders	306	0.77 (0.69–0.87)	0.79 (19.29)	0.79 (0.70)	−0.35 (−0.52)
Investigations	166	0.59 (0.50–0.68)	0.6 (46.75)	0.6 (0.51)	−0.74 (−0.96)
Infections and infestations	125	0.41 (0.34–0.49)	0.43 (102.15)	0.43 (0.36)	−1.23 (−1.48)
Musculoskeletal and connective tissue disorders	119	0.47 (0.39–0.57)	0.49 (68.30)	0.49 (0.40)	−1.04 (−1.30)
Skin and subcutaneous tissue disorders	117	0.43 (0.36–0.51)	0.44 (87.22)	0.44 (0.37)	−1.18 (−1.44)
Respiratory, thoracic and mediastinal disorders	93	0.41 (0.33–0.50)	0.42 (79.04)	0.42 (0.34)	−1.26 (−1.55)

Abbreviations: SOC, system organ class; ROR, reporting odds ratio; PRR, proportional reporting ratio; EBGM, empirical Bayes geometric mean; IC, information component; CI, confidence interval; IC025, lower limit of 95% CI, of IC; EBGM05, lower limit of 95% CI, of EBGM.

**TABLE 5:** Top 10 SOCs for donanemab by report frequency in FAERS.

SOC	Number of reports	ROR (95% CI)	PRR (χ^2^)	EBGM (EBGM05)	IC (IC025)
Nervous system disorders	520	10.16 (9.06–11.39)	6.13 (2,404.71)	6.13 (5.46)	2.62 (2.45)
Injury, poisoning and procedural complications	110	0.65 (0.53–0.79)	0.68 (19.47)	0.68 (0.56)	−0.56 (−0.84)
General disorders and administration site conditions	106	0.46 (0.38–0.56)	0.51 (61.10)	0.51 (0.42)	−0.98 (−1.26)
Psychiatric disorders	63	1.15 (0.89–1.48)	1.14 (1.18)	1.14 (0.89)	0.19 (−0.18)
Gastrointestinal disorders	60	0.6 (0.47–0.78)	0.62 (14.81)	0.62 (0.48)	−0.68 (−1.05)
Investigations	53	0.77 (0.58–1.01)	0.78 (3.64)	0.78 (0.59)	−0.37 (−0.76)
Vascular disorders	46	2.16 (1.61–2.90)	2.12 (27.60)	2.12 (1.58)	1.08 (0.62)
Skin and subcutaneous tissue disorders	44	0.66 (0.49–0.89)	0.67 (7.43)	0.67 (0.50)	−0.57 (−1.00)
Respiratory, thoracic and mediastinal disorders	38	0.68 (0.49–0.94)	0.69 (5.51)	0.69 (0.50)	−0.53 (−0.99)
Musculoskeletal and connective tissue disorders	34	0.55 (0.39–0.77)	0.56 (12.28)	0.56 (0.40)	−0.83 (−1.31)

Abbreviations: SOC, system organ class; ROR, reporting odds ratio; PRR, proportional reporting ratio; EBGM, empirical Bayes geometric mean; IC, information component; CI, confidence interval; IC025, lower limit of 95% CI, of IC; EBGM05, lower limit of 95% CI, of EBGM.

### 3.3 PT-level signal distribution

At the PT level, 63 lecanemab-positive PT signals and 34 donanemab-positive PT signals were identified. After FDR correction, 62 of the 63 lecanemab-positive PT signals and 33 of the 34 donanemab-positive PT signals remained statistically significant. The only PT signals that no longer remained statistically significant after FDR correction were sudden death for lecanemab and aphasia for donanemab; both were low-frequency signals with only three reports. Tables 6, 7 present the top 20 positive PT signals for the two drugs ranked by report frequency, together with their corresponding disproportionality signal strengths. All positive PT signals are listed in Supplementary Table S1. Ranked by report frequency, the top five positive PT signals for lecanemab were headache, ARIA-E, ARIA-H, infusion-related reaction, and chills, whereas those for donanemab were ARIA-E, ARIA-H, headache, infusion-related reaction, and cerebral haemorrhage.

**TABLE 6:** Top 20 positive preferred terms (PTs) for lecanemab by report frequency in FAERS.

PT	Number of reports	ROR (95% CI)	PRR (χ^2^)	IC025	EBGM05
Headache^a^	368	9.36 (8.41–10.41)	8.71 (2,526.51)	2.93	7.81
Amyloid related imaging abnormality-oedema/effusion^a^	297	4,092.39 (3,410.77–4,910.22)	3,838.44 (455,787.35)	7.75	1,280.15
Amyloid related imaging abnormality-microhaemorrhages and haemosiderin deposits^a^	282	4,647.32 (3,826.59–5,644.08)	4,373.49 (455,062.71)	7.69	1,329.79
Infusion related reaction^a^	194	30.31 (26.23–35.02)	29.12 (5,216.34)	4.44	24.93
Chills^a^	187	23.41 (20.21–27.11)	22.53 (3,821.39)	4.11	19.30
Fatigue^a^	163	2.69 (2.30–3.14)	2.63 (166.94)	1.15	2.25
Pyrexia^a^	123	5.05 (4.22–6.04)	4.95 (388.43)	2.00	4.13
Confusional state^a^	122	12.61 (10.53–15.10)	12.31 (1,264.75)	3.23	10.24
Dizziness^a^	110	3.39 (2.81–4.10)	3.33 (180.78)	1.43	2.76
Tremor^%^	69	7.30 (5.76–9.27)	7.21 (368.91)	2.38	5.67
Amyloid related imaging abnormalities^a^	63	3,141.63 (2,173.65–4,540.66)	3,100.28 (88,261.33)	5.48	970.31
Cerebral haemorrhage^a^	59	30.16 (23.29–39.04)	29.80 (1,623.75)	3.94	22.76
Influenza like illness^a^	57	12.93 (9.95–16.80)	12.79 (616.75)	3.02	9.80
Somnolence^%^	41	3.18 (2.34–4.32)	3.16 (60.55)	1.14	2.32
Feeling cold^%^	35	18.88 (13.52–26.35)	18.74 (583.90)	3.16	13.33
Memory impairment^%^	33	3.39 (2.41–4.78)	3.38 (55.29)	1.16	2.40
Seizure^a^	33	3.19 (2.26–4.49)	3.17 (49.21)	1.08	2.25
Dementia Alzheimer’s type^%^	31	49.34 (34.54–70.48)	49.03 (1,431.28)	3.77	33.69
Cerebral infarction^%^	24	17.73 (11.85–26.51)	17.64 (374.32)	2.82	11.72
Syncope^%^	24	3.98 (2.66–5.94)	3.96 (53.10)	1.24	2.65

^a^ PT listed in the FDA, label; %, PT, reported in clinical trials or previous literature but not listed in the label. Abbreviations: PT, preferred term; ROR, reporting odds ratio; CI, confidence interval; IC025, lower limit of 95% CI, of IC; EBGM05, lower limit of 95% CI, of EBGM.

**TABLE 7:** Top 20 positive preferred terms (PTs) for donanemab by report frequency in FAERS.

PT	Number of reports	ROR (95% CI)	PRR (χ^2^)	IC025	EBGM05
Amyloid related imaging abnormality-oedema/effusion^#^	95	2,673.58 (2,121.15–3,369.88)	2,458.96 (188,622.95)	6.18	1,576.62
Amyloid related imaging abnormality-microhaemorrhages and haemosiderin deposits^a^	81	2,459.70 (1919.82–3,151.41)	2,291.35 (151,840.45)	5.94	1,464.49
Headache^a^	72	7.26 (5.72–9.21)	6.88 (364.71)	2.32	5.42
Infusion related reaction^a^	57	36.03 (27.60–47.04)	34.34 (1841.71)	4.05	26.22
Amyloid related imaging abnormalities^a^	37	5,069.82 (3,413.69–7,529.43)	4,911.29 (123,202.94)	4.69	2,243.18
Cerebral haemorrhage^a^	32	66.82 (46.98–95.04)	65.04 (2006.01)	3.95	45.44
Flushing^a^	28	24.10 (16.56–35.08)	23.55 (603.93)	3.18	16.15
Confusional state^%^	26	10.79 (7.32–15.92)	10.58 (225.72)	2.40	7.16
Cerebral microhaemorrhage^a^	22	1771.69 (1,123.76–2,793.19)	1738.76 (32,715.08)	3.85	944.38
Brain oedema^a^	19	99.50 (63.10–156.89)	97.92 (1805.86)	3.41	61.52
Chills^a^	19	9.32 (5.92–14.68)	9.19 (138.82)	2.05	5.84
Cerebrovascular accident^%^	18	8.81 (5.53–14.03)	8.69 (122.56)	1.96	5.45
Seizure^%^	16	6.30 (3.84–10.31)	6.22 (70.26)	1.54	3.80
Back pain^%^	15	3.74 (2.25–6.23)	3.71 (29.79)	0.94	2.23
Blood pressure decreased^a^	12	10.80 (6.12–19.08)	10.70 (105.56)	1.81	6.05
Blood pressure increased^a^	12	4.10 (2.32–7.24)	4.07 (27.81)	0.91	2.30
Erythema^a^	12	3.61 (2.04–6.37)	3.58 (22.37)	0.77	2.03
Chest discomfort^a^	9	4.87 (2.53–9.38)	4.84 (27.43)	0.89	2.51
Anaphylactic reaction^a^	8	7.12 (3.55–14.26)	7.07 (41.73)	1.11	3.53
Feeling cold^%^	7	15.16 (7.21–31.90)	15.08 (91.93)	1.42	7.16

^a^ PT, listed in the FDA, label; %, PT, reported in clinical trials or previous literature but not listed in the label. Abbreviations: PT, preferred term; ROR, reporting odds ratio; CI, confidence interval; IC025, lower limit of 95% CI, of IC; EBGM05, lower limit of 95% CI, of EBGM.

**FIGURE 3:**
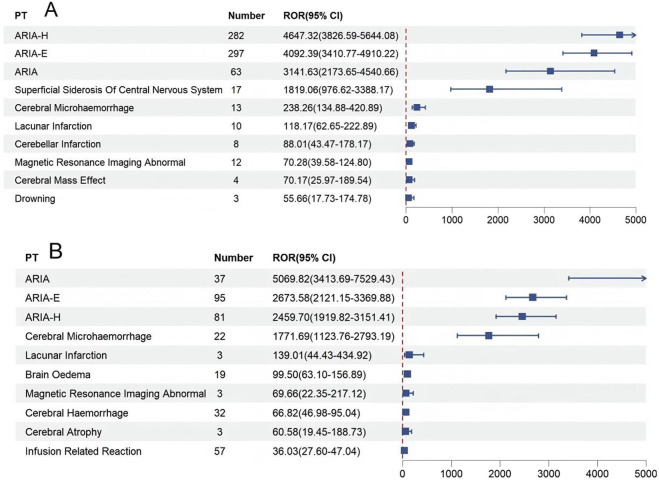
Forest plots of the top 10 positive PT signals ranked by ROR for lecanemab **(A)** and donanemab **(B)**. Error bars represent 95% confidence intervals.

Figure 3 shows the top 10 positive PT signals ranked by ROR for the two drugs. For lecanemab, the top five positive PT signals ranked by ROR were ARIA-H, ARIA-E, ARIA, superficial siderosis of central nervous system, and cerebral microhaemorrhage. For donanemab, the top five positive PT signals ranked by ROR were ARIA, ARIA-E, ARIA-H, cerebral microhaemorrhage, and lacunar infarction.

### 3.4 Shared and distinct PT signals between the two drugs

**FIGURE 4:**
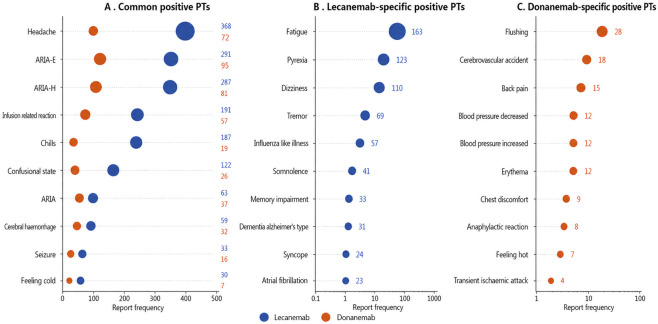
Distribution of shared and drug-specific positive PTs by report frequency for lecanemab and donanemab. **(A)** Top 10 positive PTs shared by both drugs; **(B)** Top 10 lecanemab-specific positive PTs; **(C)** Top 10 donanemab-specific positive PTs.

**FIGURE 5:**
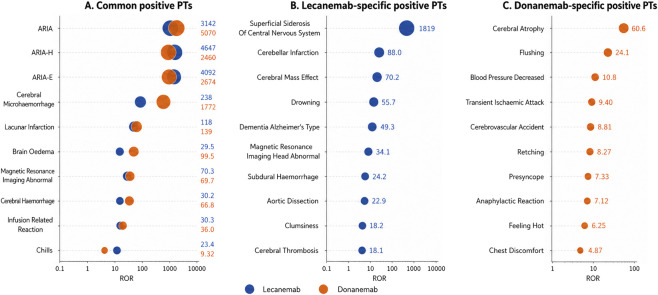
Distribution of shared and drug-specific positive PTs by ROR for lecanemab and donanemab. **(A)** Top 10 shared positive PTs; **(B)** Top 10 lecanemab-specific positive PTs; **(C)** Top 10 donanemab-specific positive PTs.

Figures 4, 5 present the distributions of shared and drug-specific positive PT signals by report frequency and signal strength. Frequently reported positive PT signals shared by both drugs were mainly concentrated in the ARIA spectrum, headache, and infusion-related reaction, whereas the strongest positive PT signals were mainly concentrated in the ARIA spectrum and cerebral microhaemorrhage. A total of 42 drug-specific positive PT signals were observed only for lecanemab. Ranked by ROR, the top five were superficial siderosis of central nervous system, cerebellar infarction, cerebral mass effect, subdural haemorrhage, and aortic dissection; ranked by report frequency, the top five were fatigue, pyrexia, dizziness, tremor, and influenza-like illness. A total of 13 drug-specific positive PT signals were observed only for donanemab. Ranked by ROR, the top five were cerebral atrophy, flushing, blood pressure decreased, transient ischaemic attack, and cerebrovascular accident; ranked by report frequency, the top five were flushing, cerebrovascular accident, back pain, blood pressure decreased, and blood pressure increased. In addition, with regard to ARIA subtypes, the disproportionality reporting signal for ARIA-H was stronger than that for ARIA-E in lecanemab reports, whereas the disproportionality reporting signal for ARIA-E was stronger than that for ARIA-H in donanemab reports.

### 3.5 Evidence stratification of positive PTs

This study further performed evidence stratification for positive PT signals associated with the two drugs. The stratification results showed that most frequently reported positive PT signals for both agents were generally consistent with existing labels or previously published evidence. However, several signals were not sufficiently covered by current labeling. Among lecanemab-associated signals, the frequently reported potential novel signals mainly included balance disorder, hallucination, and head discomfort. Among donanemab-associated signals, the frequently reported potential novel signals mainly included subarachnoid haemorrhage, magnetic resonance imaging abnormal, and cerebral atrophy. The complete stratification results for positive PT signals are presented in Supplementary Tables S2, S3.

### 3.6 Sex-stratified analysis

**FIGURE 6:**
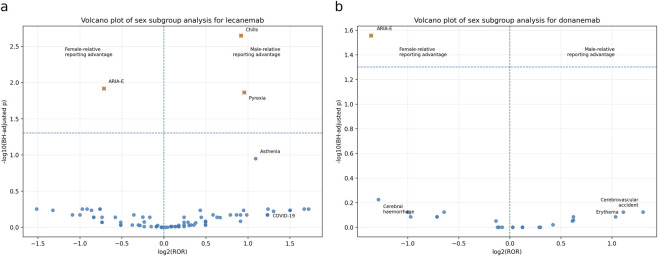
Sex-stratified analysis of positive PT signals for lecanemab and donanemab. **(A)** Volcano plot of sex‐related differences in lecanemab-associated adverse event reporting. **(B)** Volcano plot of sex-related differences in donanemab-associated adverse event reporting. The x‐axis represents log2(relative odds ratio), and the y‐axis represents ‐log10( P value). The dashed lines indicate the predefined thresholds for statistical significance.

Figure 6 shows the results of the sex-stratified analysis for the two drugs. For lecanemab, disproportionate reporting signals for asthenia, chills, COVID-19, and pyrexia were relatively higher in male reports, whereas the disproportionate reporting signal for ARIA-E was relatively higher in female reports. After FDR correction, ARIA-E, chills, and pyrexia remained statistically significant, whereas asthenia and COVID-19 no longer remained significant. For donanemab, no obvious sex-related signal difference was observed in male reports, whereas the disproportionate reporting signal for ARIA-E was relatively higher in female reports and remained statistically significant after FDR correction.

### 3.7 Sensitivity analysis

The top 10 concomitant medications excluded in the sensitivity analysis are listed in Supplementary Table S4. After excluding reports involving these concomitant medications, positive PT signals such as the ARIA spectrum, headache, cerebral haemorrhage, and infusion-related reaction were still detectable in lecanemab-related reports. In addition, potential novel reporting signals, including head discomfort and hallucination, were still observed. For donanemab, positive PT signals including the ARIA spectrum, headache, cerebral haemorrhage, chills, and infusion-related reaction were still detectable after sensitivity analysis, and potential novel reporting signals such as subarachnoid haemorrhage and cerebral atrophy were also observed. After FDR correction, all PT signals that met the positivity criteria of all four algorithms in the sensitivity analysis remained statistically significant for both lecanemab and donanemab. Detailed results of the sensitivity analysis are provided in Supplementary Tables S5, S6.

### 3.8 Comparative analysis of TTO

**FIGURE 7:**
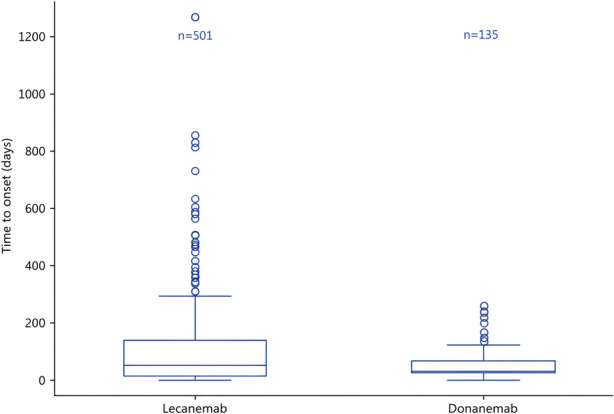
Boxplots of time‐to‐onset for lecanemab and donanemab in FAERS. The box represents the interquartile range (Q1 to Q3), the line inside the box indicates the median, whiskers denote the range of non-outlier values, and dots represent outliers.

Boxplots of TTO for the two drugs are shown in Figure 7. After deduplication by primaryid, 501 and 135 cases with calculable TTO were included for lecanemab and donanemab, respectively. The median TTO was 46 days for lecanemab and 31 days for donanemab, and the TTO distribution of lecanemab showed a wider range. Table 8 presents the Weibull distributions of onset time for lecanemab- and donanemab-related AEs. The shape parameter β for lecanemab was 0.71 (95% CI: 0.67–0.76), indicating a early failure pattern. For donanemab, β was 1.15 (95% CI: 1.01–1.33). Although this met the predefined criterion for a wear-out failure pattern, the tendency was weak because the β value was only slightly above 1.

**TABLE 8:** Time-to-onset analysis of lecanemab and donanemab related adverse events using the Weibull distribution.

Drug	​	Time-to-onset (days)		Weibull distribution				
	N	Median (IQR)	Min-max	Scale parameter		Shape parameter		Failure type
				α	95% CI	β	95% CI	
Lecanemab	501	46 (15–122)	1–1,260	79.50	69.20–90.18	0.71	0.67–0.76	Early failure
Donanemab	135	31 (27–63)	1–230	52.34	44.43–60.45	1.15	1.01–1.33	Wear-out failure

Abbreviations: IQR, interquartile range; α, scale parameter; β, shape parameter; CI, confidence interval.

## 4 Discussion

This study systematically compared the post-marketing adverse event reporting profiles of lecanemab and donanemab based on the FAERS database. These findings provide pharmacovigilance evidence that may help inform targeted safety monitoring in clinical practice. The results showed that both drugs had higher proportions of reports in female patients than in male patients, which is generally consistent with the epidemiological characteristics of AD (GBD, 2016 Disease and Injury Incidence and Prevalence Collaborators, 2017). Reports for both drugs were predominantly from the United States, which may be related to the source composition of the FAERS database and the longer period of market availability of both drugs in the United States. Lecanemab reports were mainly concentrated in 2024–2025, whereas donanemab reports were highly concentrated in 2025, which is consistent with the difference in approval timing between the two agents.

The two drugs showed shared AE patterns. At the SOC level, AEs for both drugs were mainly concentrated in nervous system disorders, which is consistent with their common mechanism as anti-Aβ monoclonal antibodies and suggests potential effects on the central nervous system (Sperling et al., 2014; Greenberg et al., 2020). At the PT level, the shared AEs for both drugs were generally consistent with the overall safety findings from pivotal clinical trials. In the pivotal Clarity AD trial of lecanemab, the most common AEs were infusion-related reactions (26.4%), ARIA-H (17.3%), ARIA-E (12.6%), and headache (11.1%) (van Dyck et al., 2023). In the TRAILBLAZER-ALZ series for donanemab, the main AEs included infusion-related reactions (8.7%), ARIA-H (31.4%), ARIA-E (24.0%), and headache (13.0%) (Zimmer et al., 2025).

The ARIA spectrum is the most representative safety concern during anti-Aβ monoclonal antibody therapy and mainly manifests as oedema or exudative changes on magnetic resonance imaging (MRI) and as microhaemorrhage or haemosiderin deposition (Hampel et al., 2023). Its mechanism may be related to reduced vascular wall stability, increased blood-brain barrier permeability, and local small-vessel injury during Aβ clearance (Walsh et al., 2022; Qiu et al., 2023). Most ARIA events are asymptomatic and may resolve after treatment interruption (Filippi et al., 2022), but some patients may develop focal neurological deficits, seizures, or even coma (Merkel et al., 2026). Apolipoprotein E ε4 (APOE ε4), particularly in the homozygous state, is a shared risk factor for ARIA associated with both drugs (Cohe et al., 2023; Evans et al., 2023). Cerebral microhaemorrhage showed a strong disproportionality signal for both drugs. Because cerebral microhaemorrhage is an important manifestation of ARIA-H, this finding suggests that haemorrhagic cerebrovascular AEs should be closely monitored during real-world treatment with both drugs, especially in APOE ε4 carriers, in patients with prior cerebral microbleeds, or in those receiving anticoagulant therapy. In addition to ARIA, infusion-related reaction was also a common AE for both drugs and may be associated with acute immune-mediated effects during early monoclonal antibody treatment. In clinical practice, close monitoring should be strengthened during the early phase of treatment, particularly at the first infusion, and prophylactic corticosteroids or antihistamines may be considered when necessary (Noguchi-Shinohara et al., 2025; U.S. Food and Drug Administration, 2025).

Despite the consistency in overall reporting patterns, notable differences were also observed between the two drugs, particularly in the distribution of ARIA subtypes. These differences may be partly related to the distinct Aβ species targeted by the two agents and their clearance kinetics, as lecanemab mainly targets soluble Aβ protofibrils, whereas donanemab preferentially recognizes deposited plaque-related antigens (Gueorguieva et al., 2023; Johannesson et al., 2024). However, the ARIA subtype-related differences observed in this study may not be fully consistent with previous clinical trial findings. FAERS differs substantially from clinical trials in terms of data sources, population composition, monitoring procedures, and reporting mechanisms. FAERS reflects spontaneous real-world reporting, with more heterogeneous case sources, non-standardized reporting criteria, and a lack of uniform imaging assessment, rather than systematically collected clinical outcomes. Therefore, the observed ARIA subtype distribution may be influenced by reporting behavior, monitoring intensity, approval timing, reporting maturity, and patient characteristics, which may partly explain the discrepancy between our findings and previous clinical trial results. In addition, several factors that may substantially influence ARIA risk, including disease stage, APOE genotype, baseline MRI burden, and anticoagulant use, are not systematically available in FAERS and may confound the observed reporting patterns. FAERS also lacks standardized information on ARIA severity. Therefore, the observed ARIA subtype-related differences should be interpreted as pharmacovigilance reporting patterns rather than evidence of true subtype-specific clinical risk differences between the two drugs.

Drug-specific positive PTs also showed distinct reporting signal patterns between the two agents. Overall, lecanemab-specific reporting signals were more frequently related to neurological symptoms and cerebrovascular events, whereas donanemab-specific reporting signals were more often associated with haemodynamic fluctuations and ischemic cerebrovascular events. For lecanemab, dizziness and somnolence were among the relatively frequently reported drug-specific signals. Previous studies have suggested that Aβ clearance during lecanemab treatment may affect brain regions involved in sleep-wake regulation and alter the local microenvironment and neuroinflammatory status, which may partly manifest as non-specific neurological symptoms such as somnolence (Özcan et al., 2020; Zielinski and Gibbons, 2022). However, because these events were less frequently reported in previous clinical trials, they should currently be regarded as pharmacovigilance reporting signals requiring further attention. Although superficial siderosis was reported infrequently, its signal strength was relatively high, suggesting that imaging findings related to haemosiderin deposition over the brain surface may warrant attention during subsequent clinical monitoring. For donanemab, flushing and blood pressure fluctuation-related signals showed relatively high report counts and signal strength, suggesting that vascular reactions may occur during infusion. Vital signs may need to be monitored carefully during infusion, particularly in patients with cardiovascular or cerebrovascular comorbidities or baseline blood pressure instability.

Ischemic cerebrovascular reporting signals for both drugs should be interpreted with particular caution. In lecanemab-related reports, cerebral infarction was identified as a positive reporting signal, whereas transient ischaemic attack was observed among donanemab-related signals. However, because FAERS is a spontaneous reporting database, these findings cannot establish a causal relationship between anti-Aβ monoclonal antibody therapy and ischemic cerebrovascular events. In elderly patients with AD, ischemic cerebrovascular events may be influenced by underlying vascular comorbidities, baseline cerebrovascular risk, antithrombotic therapy, frailty, and other unmeasured clinical factors. Therefore, these ischemic cerebrovascular events should be regarded as pharmacovigilance reporting signals rather than confirmatory evidence that they were caused by lecanemab or donanemab.

In lecanemab-related reports, hallucination and balance disorder were observed as potential novel signals. However, given the limited number of reports and the possibility that hallucination may reflect Alzheimer’s disease-related neuropsychiatric symptoms or disease progression, and that balance disorder may also be associated with fall risk, cerebrovascular comorbidities, and frailty in elderly patients, these findings should be interpreted cautiously. Similarly, the donanemab-associated signals of subarachnoid haemorrhage and cerebral atrophy should not be overinterpreted. Subarachnoid haemorrhage may be influenced by underlying cerebrovascular risk, concomitant medications, and unmeasured confounding factors, whereas cerebral atrophy may reflect the natural progression of Alzheimer’s disease, differences in imaging surveillance intensity, or reporting bias. Overall, these signals should be regarded as hypothesis-generating pharmacovigilance signals rather than confirmed new adverse reactions.

Sex-stratified analyses suggested that some PT-level reporting patterns differed between female and male reports. After FDR correction, ARIA-E showed a relatively higher reporting signal in female reports for both lecanemab and donanemab, whereas chills and pyrexia remained relatively more prominent in male reports for lecanemab. However, these findings should be interpreted cautiously because spontaneous reporting data may be influenced by monitoring intensity, reporting behavior, and baseline clinical characteristics. In particular, sex information was missing in 24.7% of donanemab-related reports, which may weaken the reliability and stability of the sex-stratified analysis for donanemab and limit the interpretation of apparent sex-related reporting differences. In the sensitivity analysis excluding reports involving the top 10 concomitant medications, most core reporting signals remained detectable for both drugs, suggesting that these signals were not solely driven by the most frequently reported concomitant medications.

The TTO and WSP analyses suggested differences in the temporal reporting patterns of AEs between the two drugs. Lecanemab was classified as showing an early failure pattern, suggesting that reported AEs tended to occur earlier after treatment initiation, which may be partly consistent with the clinical observation that infusion-related reactions are more commonly reported during the initial stage of anti-Aβ monoclonal antibody therapy. Although donanemab was classified as showing a wear-out failure pattern, its β value was only slightly above 1, and the number of reports with calculable TTO was limited within a relatively short observation window. Therefore, this result should be interpreted as a possible tendency toward temporal accumulation in reporting rather than a definite late high-risk pattern. Differences in TTO patterns between the two drugs may also have been influenced by approval timing, monitoring intensity, incomplete date reporting, and other reporting biases inherent to spontaneous reporting systems. Accordingly, these findings should be interpreted as descriptive pharmacovigilance signals rather than definitive time-dependent risk patterns.

Several limitations of this study should be acknowledged. First, direct comparisons between lecanemab and donanemab should be interpreted with caution because of differences in approval timing, market exposure windows, reporting periods, and cumulative drug utilization. Lecanemab received FDA approval in July 2023, whereas donanemab was approved in July 2024. Accordingly, the two drugs differed in reporting maturity, cumulative prescriptions, and observable follow-up duration, which may have influenced report volume, event composition, disproportionality findings, and TTO distributions. In particular, donanemab-related reports were concentrated within a relatively short time window, making direct comparisons susceptible to differences in market exposure and reporting opportunities. Therefore, the present findings should be interpreted as comparative reporting signals rather than evidence of true comparative safety risks. Second, FAERS does not provide exposure denominators, prescription numbers, or the total number of treated patients; therefore, incidence, prevalence, absolute risk, and relative risk cannot be estimated. As a spontaneous reporting system, FAERS is inherently subject to underreporting, selective reporting, duplicate reporting, and incomplete information, precluding causal inference between drugs and specific adverse events. Moreover, differences in reporter composition, such as the proportions of reports submitted by consumers, physicians, and other healthcare professionals, may also have influenced the observed reporting patterns. In addition, several important clinical variables that may substantially influence ARIA risk, including disease stage, APOE genotype, baseline MRI burden, anticoagulant use, and ARIA severity, are not systematically available in FAERS and may confound the observed reporting patterns. Although a sensitivity analysis excluding the top 10 concomitant medications was performed, residual confounding from other concomitant drugs, drug combinations, unreported medications, or unmeasured clinical factors may still remain. Third, missing demographic information may have affected the reliability of subgroup analyses, particularly for donanemab-related reports. The proportion of missing sex data for donanemab was 24.7%, which may weaken the reliability and stability of the sex-stratified analysis for donanemab. In addition, age information was missing in 54.4% of donanemab-related reports, which may limit the stability and interpretation of age-related findings. Furthermore, TTO information was available for only 501 lecanemab reports and 135 donanemab reports. Therefore, the TTO and Weibull analyses, particularly for donanemab, may be statistically unstable and should be interpreted cautiously. Finally, this study was based solely on the FAERS database, and external validation using other pharmacovigilance databases could not be completed. Therefore, database-specific reporting bias may still exist.

## 5 Conclusion

Based on the FAERS database, this study systematically compared the real-world adverse event reporting profiles of lecanemab and donanemab using four signal detection algorithms. The results showed that the most prominent AEs for both drugs were nervous system-related events, with frequently reported AEs mainly including the ARIA spectrum, headache, and infusion-related reaction. The ARIA spectrum and cerebral microhaemorrhage showed strong disproportionality signals with both drugs. At the same time, certain differences were observed between the two drugs in the distribution of ARIA subtypes, drug-specific reporting signals, and temporal patterns of event occurrence. Sex-stratified analysis and sensitivity analysis based on concomitant medications further supplemented and supported the main findings. These results may provide real-world pharmacovigilance evidence to support individualized treatment considerations and targeted risk monitoring in clinical practice, but they should not be interpreted as causal associations, incidence estimates, or definitive comparative safety risks. Future studies based on multiple databases and longer post-marketing observation periods are warranted to further validate and extend these findings.

## Data Availability

The datasets presented in this study can be found in online repositories. The names of the repository/repositories and accession number(s) can be found in the article/Supplementary Material.
