# Supplementary material for: Comparison of safety of lecanemab and donanemab: a real-world disproportionality analysis using the FDA adverse event reporting system
Source: Front Pharmacol. 2026 Jun 22;17:1868789. doi: 10.3389/fphar.2026.1868789 (PMC13333721; doi:10.3389/fphar.2026.1868789)
Supplement: Supplementary file 1 [file Table1.docx]

**Supplementary Table S1.** Lecanemab: 63 positive PTs with unified symbol annotation

| **No.** | **PT** | **a** | **ROR (95% CI)** | **PRR (χ²)** | **IC025** | **EBGM05** |
| --- | --- | --- | --- | --- | --- | --- |
| 1 | Headache# | 368 | 9.36 ( 8.41 - 10.41 ) | 8.71 ( 2526.51 ) | 2.93 | 7.81 |
| 2 | ARIA-E# | 297 | 4092.39 ( 3410.77 - 4910.22 ) | 3838.44 ( 455787.35 ) | 7.75 | 1280.15 |
| 3 | ARIA-H# | 282 | 4647.32 ( 3826.59 - 5644.08 ) | 4373.49 ( 455062.71 ) | 7.69 | 1329.79 |
| 4 | Infusion related reaction# | 194 | 30.31 ( 26.23 - 35.02 ) | 29.12 ( 5216.34 ) | 4.44 | 24.93 |
| 5 | Chills# | 187 | 23.41 ( 20.21 - 27.11 ) | 22.53 ( 3821.39 ) | 4.11 | 19.3 |
| 6 | Fatigue# | 163 | 2.69 ( 2.3 - 3.14 ) | 2.63 ( 166.94 ) | 1.15 | 2.25 |
| 7 | Pyrexia# | 123 | 5.05 ( 4.22 - 6.04 ) | 4.95 ( 388.43 ) | 2.0 | 4.13 |
| 8 | Confusional state# | 122 | 12.61 ( 10.53 - 15.1 ) | 12.31 ( 1264.75 ) | 3.23 | 10.24 |
| 9 | Dizziness# | 110 | 3.39 ( 2.81 - 4.1 ) | 3.33 ( 180.78 ) | 1.43 | 2.76 |
| 10 | Tremor% | 69 | 7.3 ( 5.76 - 9.27 ) | 7.21 ( 368.91 ) | 2.38 | 5.67 |
| 11 | ARIA# | 63 | 3141.63 ( 2173.65 - 4540.66 ) | 3100.28 ( 88261.33 ) | 5.48 | 970.31 |
| 12 | Cerebral haemorrhage# | 59 | 30.16 ( 23.29 - 39.04 ) | 29.8 ( 1623.75 ) | 3.94 | 22.76 |
| 13 | Influenza like illness# | 57 | 12.93 ( 9.95 - 16.8 ) | 12.79 ( 616.75 ) | 3.02 | 9.8 |
| 14 | Somnolence% | 41 | 3.18 ( 2.34 - 4.32 ) | 3.16 ( 60.55 ) | 1.14 | 2.32 |
| 15 | Feeling cold% | 35 | 18.88 ( 13.52 - 26.35 ) | 18.74 ( 583.9 ) | 3.16 | 13.33 |
| 16 | Memory impairment% | 33 | 3.39 ( 2.41 - 4.78 ) | 3.38 ( 55.29 ) | 1.16 | 2.4 |
| 17 | Seizure# | 33 | 3.19 ( 2.26 - 4.49 ) | 3.17 ( 49.21 ) | 1.08 | 2.25 |
| 18 | Dementia alzheimer's type% | 31 | 49.34 ( 34.54 - 70.48 ) | 49.03 ( 1431.28 ) | 3.77 | 33.69 |
| 19 | Cerebral infarction% | 24 | 17.73 ( 11.85 - 26.51 ) | 17.64 ( 374.32 ) | 2.82 | 11.72 |
| 20 | Syncope% | 24 | 3.98 ( 2.66 - 5.94 ) | 3.96 ( 53.1 ) | 1.24 | 2.65 |
| 21 | Brain oedema# | 23 | 29.49 ( 19.53 - 44.53 ) | 29.35 ( 622.9 ) | 3.15 | 19.23 |
| 22 | Atrial fibrillation# | 23 | 3.5 ( 2.32 - 5.28 ) | 3.49 ( 40.87 ) | 1.07 | 2.31 |
| 23 | Balance disorder* | 22 | 4.11 ( 2.7 - 6.24 ) | 4.09 ( 51.36 ) | 1.25 | 2.69 |
| 24 | Cognitive disorder% | 21 | 6.43 ( 4.19 - 9.88 ) | 6.41 ( 95.63 ) | 1.74 | 4.16 |
| 25 | Lethargy% | 20 | 6.01 ( 3.87 - 9.33 ) | 5.99 ( 82.98 ) | 1.64 | 3.85 |
| 26 | Aphasia# | 19 | 10.51 ( 6.69 - 16.51 ) | 10.47 ( 162.22 ) | 2.18 | 6.64 |
| 27 | Hallucination* | 19 | 3.32 ( 2.11 - 5.21 ) | 3.31 ( 30.58 ) | 0.92 | 2.11 |
| 28 | Superficial siderosis of central nervous system# | 17 | 1819.06 ( 976.62 - 3388.17 ) | 1812.6 ( 18017.7 ) | 3.33 | 569.87 |
| 29 | Disorientation% | 17 | 8.87 ( 5.5 - 14.29 ) | 8.84 ( 117.85 ) | 1.94 | 5.47 |
| 30 | Subdural haematoma% | 15 | 22.41 ( 13.47 - 37.28 ) | 22.34 ( 303.2 ) | 2.53 | 13.32 |
| 31 | Brain fog% | 14 | 3.82 ( 2.26 - 6.45 ) | 3.81 ( 28.99 ) | 0.93 | 2.25 |
| 32 | Cerebral microhaemorrhage# | 13 | 238.26 ( 134.88 - 420.89 ) | 237.62 ( 2802.86 ) | 2.92 | 123.13 |
| 33 | Magnetic resonance imaging abnormal# | 12 | 70.28 ( 39.58 - 124.8 ) | 70.11 ( 795.7 ) | 2.65 | 38.44 |
| 34 | Lacunar infarction% | 10 | 118.17 ( 62.65 - 222.89 ) | 117.92 ( 1108.28 ) | 2.44 | 59.79 |
| 35 | Ischaemic stroke% | 10 | 10.32 ( 5.54 - 19.22 ) | 10.3 ( 83.67 ) | 1.6 | 5.51 |
| 36 | Head discomfort* | 10 | 6.73 ( 3.62 - 12.53 ) | 6.72 ( 48.58 ) | 1.27 | 3.6 |
| 37 | Head injury% | 10 | 4.41 ( 2.37 - 8.21 ) | 4.41 ( 26.31 ) | 0.88 | 2.37 |
| 38 | Cerebellar infarction* | 8 | 88.01 ( 43.47 - 178.17 ) | 87.86 ( 664.19 ) | 2.06 | 41.97 |
| 39 | Status epilepticus# | 8 | 11.18 ( 5.58 - 22.4 ) | 11.16 ( 73.7 ) | 1.42 | 5.55 |
| 40 | Generalised tonic-clonic seizure* | 8 | 5.69 ( 2.84 - 11.39 ) | 5.68 ( 30.78 ) | 0.94 | 2.83 |
| 41 | Anger% | 8 | 5.03 ( 2.51 - 10.08 ) | 5.03 ( 25.77 ) | 0.83 | 2.51 |
| 42 | Subarachnoid haemorrhage* | 7 | 10.03 ( 4.77 - 21.07 ) | 10.01 ( 56.57 ) | 1.21 | 4.75 |
| 43 | Body temperature increased# | 7 | 5.28 ( 2.51 - 11.09 ) | 5.27 ( 24.19 ) | 0.76 | 2.51 |
| 44 | Mental status changes% | 7 | 4.87 ( 2.32 - 10.24 ) | 4.87 ( 21.49 ) | 0.69 | 2.31 |
| 45 | Infusion site extravasation* | 6 | 10.52 ( 4.72 - 23.47 ) | 10.51 ( 51.42 ) | 1.06 | 4.69 |
| 46 | Abnormal dreams% | 6 | 5.28 ( 2.37 - 11.78 ) | 5.28 ( 20.77 ) | 0.62 | 2.36 |
| 47 | Magnetic resonance imaging head abnormal* | 5 | 34.06 ( 14.09 - 82.35 ) | 34.03 ( 158.19 ) | 1.2 | 13.9 |
| 48 | Incontinence% | 5 | 7.27 ( 3.02 - 17.49 ) | 7.26 ( 26.92 ) | 0.65 | 3.01 |
| 49 | Dizziness postural* | 5 | 5.66 ( 2.35 - 13.61 ) | 5.65 ( 19.1 ) | 0.49 | 2.34 |
| 50 | Cerebral mass effect* | 4 | 70.17 ( 25.97 - 189.54 ) | 70.11 ( 265.23 ) | 0.93 | 25.27 |
| 51 | Aortic dissection* | 4 | 22.92 ( 8.56 - 61.35 ) | 22.9 ( 83.03 ) | 0.79 | 8.48 |
| 52 | Haemorrhagic stroke% | 4 | 10.12 ( 3.79 - 27.03 ) | 10.11 ( 32.73 ) | 0.55 | 3.77 |
| 53 | Drowning* | 3 | 55.66 ( 17.73 - 174.78 ) | 55.63 ( 157.52 ) | 0.46 | 17.35 |
| 54 | Subdural haemorrhage% | 3 | 24.23 ( 7.77 - 75.56 ) | 24.22 ( 66.15 ) | 0.38 | 7.7 |
| 55 | Clumsiness* | 3 | 18.25 ( 5.86 - 56.82 ) | 18.23 ( 48.53 ) | 0.33 | 5.82 |
| 56 | Cerebral thrombosis* | 3 | 18.12 ( 5.82 - 56.42 ) | 18.11 ( 48.14 ) | 0.33 | 5.78 |
| 57 | Neuropsychological symptoms* | 3 | 13.04 ( 4.19 - 40.57 ) | 13.03 ( 33.16 ) | 0.25 | 4.17 |
| 58 | Fibrin D-dimer increased% | 3 | 11.59 ( 3.73 - 36.03 ) | 11.58 ( 28.87 ) | 0.22 | 3.71 |
| 59 | Formication% | 3 | 10.05 ( 3.23 - 31.25 ) | 10.05 ( 24.35 ) | 0.17 | 3.22 |
| 60 | Infusion site bruising* | 3 | 9.46 ( 3.04 - 29.4 ) | 9.45 ( 22.6 ) | 0.15 | 3.03 |
| 61 | Screaming% | 3 | 8.39 ( 2.7 - 26.09 ) | 8.39 ( 19.47 ) | 0.11 | 2.69 |
| 62 | Myocardial ischaemia* | 3 | 7.35 ( 2.37 - 22.84 ) | 7.35 ( 16.4 ) | 0.06 | 2.36 |
| 63 | Sudden death* | 3 | 6.82 ( 2.2 - 21.19 ) | 6.82 ( 14.86 ) | 0.03 | 2.19 |

**Supplementary Table S2.** Donanemab: 34 positive PTs with unified symbol annotation

| **No.** | **PT** | **a** | **ROR (95% CI)** | **PRR (χ²)** | **IC025** | **EBGM05** |
| --- | --- | --- | --- | --- | --- | --- |
| 1 | ARIA-E# | 95 | 2673.58 ( 2121.15 - 3369.88 ) | 2458.96 ( 188622.95 ) | 6.18 | 1576.62 |
| 2 | ARIA-H# | 81 | 2459.7 ( 1919.82 - 3151.41 ) | 2291.35 ( 151840.45 ) | 5.94 | 1464.49 |
| 3 | Headache# | 72 | 7.26 ( 5.72 - 9.21 ) | 6.88 ( 364.71 ) | 2.32 | 5.42 |
| 4 | Infusion related reaction# | 57 | 36.03 ( 27.6 - 47.04 ) | 34.34 ( 1841.71 ) | 4.05 | 26.22 |
| 5 | ARIA# | 37 | 5069.82 ( 3413.69 - 7529.43 ) | 4911.29 ( 123202.94 ) | 4.69 | 2243.18 |
| 6 | Cerebral haemorrhage# | 32 | 66.82 ( 46.98 - 95.04 ) | 65.04 ( 2006.01 ) | 3.95 | 45.44 |
| 7 | Flushing# | 28 | 24.1 ( 16.56 - 35.08 ) | 23.55 ( 603.93 ) | 3.18 | 16.15 |
| 8 | Confusional state% | 26 | 10.79 ( 7.32 - 15.92 ) | 10.58 ( 225.72 ) | 2.4 | 7.16 |
| 9 | Cerebral microhaemorrhage# | 22 | 1771.69 ( 1123.76 - 2793.19 ) | 1738.76 ( 32715.08 ) | 3.85 | 944.38 |
| 10 | Brain oedema# | 19 | 99.5 ( 63.1 - 156.89 ) | 97.92 ( 1805.86 ) | 3.41 | 61.52 |
| 11 | Chills# | 19 | 9.32 ( 5.92 - 14.68 ) | 9.19 ( 138.82 ) | 2.05 | 5.84 |
| 12 | Cerebrovascular accident% | 18 | 8.81 ( 5.53 - 14.03 ) | 8.69 ( 122.56 ) | 1.96 | 5.45 |
| 13 | Seizure% | 16 | 6.3 ( 3.84 - 10.31 ) | 6.22 ( 70.26 ) | 1.54 | 3.8 |
| 14 | Back pain% | 15 | 3.74 ( 2.25 - 6.23 ) | 3.71 ( 29.79 ) | 0.94 | 2.23 |
| 15 | Blood pressure decreased# | 12 | 10.8 ( 6.12 - 19.08 ) | 10.7 ( 105.56 ) | 1.81 | 6.05 |
| 16 | Blood pressure increased# | 12 | 4.1 ( 2.32 - 7.24 ) | 4.07 ( 27.81 ) | 0.91 | 2.3 |
| 17 | Erythema# | 12 | 3.61 ( 2.04 - 6.37 ) | 3.58 ( 22.37 ) | 0.77 | 2.03 |
| 18 | Chest discomfort# | 9 | 4.87 ( 2.53 - 9.38 ) | 4.84 ( 27.43 ) | 0.89 | 2.51 |
| 19 | Anaphylactic reaction# | 8 | 7.12 ( 3.55 - 14.26 ) | 7.07 ( 41.73 ) | 1.11 | 3.53 |
| 20 | Feeling cold% | 7 | 15.16 ( 7.21 - 31.9 ) | 15.08 ( 91.93 ) | 1.42 | 7.16 |
| 21 | Feeling hot% | 7 | 6.25 ( 2.97 - 13.14 ) | 6.22 ( 30.66 ) | 0.89 | 2.96 |
| 22 | Disorientation% | 6 | 12.65 ( 5.67 - 28.24 ) | 12.6 ( 64 ) | 1.15 | 5.64 |
| 23 | Cognitive disorder% | 6 | 7.42 ( 3.33 - 16.56 ) | 7.39 ( 33.16 ) | 0.85 | 3.31 |
| 24 | Subarachnoid haemorrhage* | 4 | 23.19 ( 8.68 - 61.95 ) | 23.11 ( 84.44 ) | 0.8 | 8.63 |
| 25 | Cerebral infarction% | 4 | 11.87 ( 4.44 - 31.69 ) | 11.83 ( 39.62 ) | 0.61 | 4.43 |
| 26 | Transient ischaemic attack% | 4 | 9.4 ( 3.52 - 25.11 ) | 9.38 ( 29.91 ) | 0.51 | 3.51 |
| 27 | Lacunar infarction% | 3 | 139.01 ( 44.43 - 434.92 ) | 138.66 ( 404.6 ) | 0.51 | 43.74 |
| 28 | Magnetic resonance imaging abnormal* | 3 | 69.66 ( 22.35 - 217.12 ) | 69.49 ( 201.16 ) | 0.49 | 22.15 |
| 29 | Cerebral atrophy* | 3 | 60.58 ( 19.45 - 188.73 ) | 60.43 ( 174.33 ) | 0.48 | 19.29 |
| 30 | Subdural haematoma* | 3 | 18 ( 5.79 - 55.94 ) | 17.95 ( 47.95 ) | 0.33 | 5.77 |
| 31 | Ischaemic stroke% | 3 | 12.5 ( 4.02 - 38.83 ) | 12.47 ( 31.62 ) | 0.24 | 4.01 |
| 32 | Retching% | 3 | 8.27 ( 2.66 - 25.71 ) | 8.26 ( 19.12 ) | 0.11 | 2.66 |
| 33 | Presyncope* | 3 | 7.33 ( 2.36 - 22.76 ) | 7.31 ( 16.33 ) | 0.06 | 2.35 |
| 34 | Aphasia% | 3 | 6.68 ( 2.15 - 20.76 ) | 6.67 ( 14.45 ) | 0.02 | 2.15 |

**Supplementary Table S3.** Evidence stratification of positive preferred term signals for lecanemab and donanemab

| Evidence tier | Lecanemab | Donanemab |
| --- | --- | --- |
| Label listed | Headache | ARIA-E |
|  | ARIA-E | ARIA-H |
|  | ARIA-H | ARIA |
|  | Infusion related reaction | Brain oedema |
|  | Chills | Cerebral microhaemorrhage |
|  | Fatigue | Cerebral haemorrhage |
|  | Pyrexia | Headache |
|  | Confusional state | Infusion related reaction |
|  | Dizziness | Chills |
|  | ARIA | Flushing |
|  | Cerebral haemorrhage | Erythema |
|  | Influenza like illness | Blood pressure increased |
|  | Seizure | Blood pressure decreased |
|  | Brain oedema | Chest discomfort |
|  | Atrial fibrillation | Anaphylactic reaction |
|  | Aphasia |  |
|  | Superficial siderosis of central nervous system |  |
|  | Cerebral microhaemorrhage |  |
|  | Magnetic resonance imaging abnormal |  |
|  | Status epilepticus |  |
|  | Body temperature increased |  |
| Trial/literature (not in label) | Tremor | Confusional state |
|  | Somnolence | Disorientation |
|  | Feeling cold | Cognitive disorder |
|  | Memory impairment | Seizure |
|  | Dementia alzheimer's type | Aphasia |
|  | Syncope | Cerebrovascular accident |
|  | Cerebral infarction | Transient ischaemic attack |
|  | Cognitive disorder | Ischaemic stroke |
|  | Lethargy | Cerebral infarction |
|  | Disorientation | Lacunar infarction |
|  | Subdural haematoma | Back pain |
|  | Brain fog | Feeling cold |
|  | Head injury | Feeling hot |
|  | Ischaemic stroke | Retching |
|  | Lacunar infarction |  |
|  | Anger |  |
|  | Mental status changes |  |
|  | Abnormal dreams |  |
|  | Incontinence |  |
|  | Haemorrhagic stroke |  |
|  | Formication |  |
|  | Screaming |  |
|  | Subdural haemorrhage |  |
|  | Fibrin D-dimer increased |  |
| Potential new signal | Balance disorder | Subarachnoid haemorrhage |
|  | Hallucination | Subdural haematoma |
|  | Head discomfort | Cerebral atrophy |
|  | Cerebellar infarction | Presyncope |
|  | Generalised tonic-clonic seizure | Magnetic resonance imaging abnormal |
|  | Subarachnoid haemorrhage |  |
|  | Infusion site extravasation |  |
|  | Dizziness postural |  |
|  | Magnetic resonance imaging head abnormal |  |
|  | Aortic dissection |  |
|  | Cerebral mass effect |  |
|  | Drowning |  |
|  | Sudden death |  |
|  | Clumsiness |  |
|  | Infusion site bruising |  |
|  | Cerebral thrombosis |  |
|  | Myocardial ischaemia |  |
|  | Neuropsychological symptoms |  |

**Supplementary Table S4.** Top 10 concomitant drugs reported with lecanemab and donanemab

| **Rank** | **Lecanemab concomitant drug** | **n** | **Drug class** | **Donanemab concomitant drug** | **n** | **Drug class** |
| --- | --- | --- | --- | --- | --- | --- |
| 1 | Donepezil | 170 | Anti-dementia drug | Donepezil | 31 | Anti-dementia drug |
| 2 | Aspirin | 116 | Antiplatelet agent | Tylenol | 22 | Analgesic/antipyretic |
| 3 | Memantine | 108 | Anti-dementia drug | Aspirin | 20 | Antiplatelet agent |
| 4 | Aricept | 88 | Anti-dementia drug | Benadryl | 19 | Antihistamine |
| 5 | Atorvastatin | 83 | Lipid-lowering drug | Memantine | 18 | Anti-dementia drug |
| 6 | Amlodipine | 73 | Antihypertensive drug | Amlodipine | 14 | Antihypertensive drug |
| 7 | Levothyroxine | 55 | Thyroid hormone | Atorvastatin | 10 | Lipid-lowering drug |
| 8 | Lisinopril | 50 | Antihypertensive drug | Acetaminophen | 9 | Analgesic/antipyretic |
| 9 | Vitamins | 50 | Supplement | Aricept | 9 | Anti-dementia drug |
| 10 | Rosuvastatin | 49 | Lipid-lowering drug | Levothyroxine | 9 | Thyroid hormone |

**Supplementary Table S5.** Established and potential novel signals retained after concomitant-drug sensitivity analysis for lecanemab

| **Signal category** | **SOC** | **PT retained after sensitivity analysis** |
| --- | --- | --- |
| Established signals | Nervous system disorders | Amyloid related imaging abnormalities; Amyloid related imaging abnormality-oedema/effusion; Amyloid related imaging abnormality-microhaemorrhages and haemosiderin deposits; Headache; Dizziness; Tremor; Cerebral haemorrhage; Somnolence; Seizure; Syncope; Cerebral infarction; Brain oedema |
|  | General disorders and administration site conditions | Infusion related reaction; Chills; Fatigue; Pyrexia; Influenza like illness |
|  | Psychiatric disorders | Confusional state |
| Potential novel signals | Nervous system disorders | Head discomfort |
|  | Psychiatric disorders | Hallucination |

Note: Signals were grouped into established signals and potential novel signals based on the evidence classification used in the manuscript. SOC classification was assigned according to the corresponding MedDRA System Organ Class.

**Supplementary Table S6.** Established and potential novel signals retained after concomitant-drug sensitivity analysis for donanemab, classified by SOC

| **Category** | **SOC** | **PT retained after sensitivity analysis** |
| --- | --- | --- |
| Established signals | Nervous system disorders | Amyloid related imaging abnormalities; Amyloid related imaging abnormality-oedema/effusion; Amyloid related imaging abnormality-microhaemorrhages and haemosiderin deposits; Headache; Cerebral haemorrhage; Cerebral microhaemorrhage; Brain oedema; Seizure |
|  | Injury, poisoning and procedural complications | Infusion related reaction |
|  | General disorders and administration site conditions | Chills |
|  | Psychiatric disorders | Confusional state |
| Potential novel signals | Nervous system disorders | Subarachnoid haemorrhage; Cerebral atrophy |
|  | Investigations | Magnetic resonance imaging abnormal |
